# MiRNA based tumor mutation burden diagnostic and prognostic prediction models for endometrial cancer

**DOI:** 10.1080/21655979.2021.1947940

**Published:** 2021-07-12

**Authors:** Nan Lu, Jinhui Liu, Chengjian Ji, Yichun Wang, Zhipeng Wu, Shuning Yuan, Yan Xing, Feiyang Diao

**Affiliations:** a Department of Reproduction, The First Affiliated Hospital with Nanjing Medical University, Nanjing, China; b Department of Gynecology, The First Affiliated Hospital of Nanjing Medical University, Nanjing, China; c Department of Urology, The First Affiliated Hospital of Nanjing Medical University, Nanjing, China; d Department of Urology, The Affiliated Sir Run Run Hospital of Nanjing Medical University, Nanjing, Jiangsu Province, China

**Keywords:** UCEC, TMB, miRNAs, prognosis signature, immunotherapy

## Abstract

Uterus Corpus Endometrial cancer (UCEC) is the sixth most common malignant tumor worldwide. In this research, we identified diagnostic and prognostic biomarkers to reflect patients’ immune microenvironment and prognostic. Various data of UCEC patients from the TCGA database were obtained. Firstly, patients were divided into a high tumor mutation burden (TMB) level group and a low TMB level group according to the level of TMB. Then, differentially expressed miRNAs between the two groups were obtained. LASSO logistic regression analysis was used to construct a diagnostic model to predict the level of TMB. Univariate, multivariate, and LASSO regression analysis were used to construct a prognostic risk signature (PRS) to predict the prognosis of UCEC patients. Twenty-one miRNAs were used to construct a diagnostic model for predicting TMB levels. The AUC values of ROC curves for 21-miRNA-based diagnostic models were 0.911 in the training set, 0.827 in the test set, and 0.878 in the entire set. This diagnostic model showed positive correlation with TMB, PDL1 expression, and the infiltration of immune cells. In addition, three prognostic miRNAs were finally used to construct the PRS. The PRS was related to the expression of multiple immune checkpoints and the infiltration of multiple immune cells. Furthermore, the PRS can also reflect the response to some commonly used chemotherapy regimens. We have established a miRNA-based diagnostic model and a prognostic model that can predict the prognosis of UCEC patients and their response to chemotherapy and immunotherapy, thus providing valuable information on the choice of treatment regimen.

## Graphical Abstract

**Figure:**
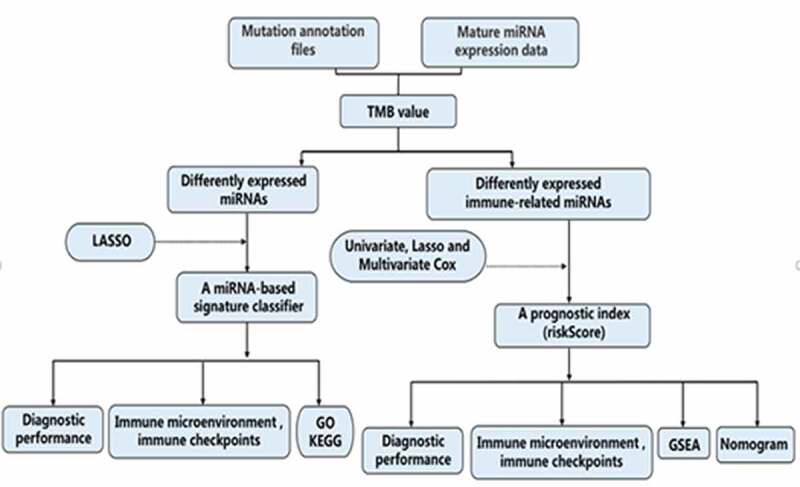

## Introduction

Uterus corpus endometrial cancer (UCEC) is one of the most common malignant tumors of the female reproductive system. The morbidity and mortality of UCEC have always been at a high level, and there is a trend of continuous growth [1],[1,1]. Currently, there are two methods for typing endometrial cancer. Bokhman proposed in 1983 that UCEC can be classified into type I (estrogen-dependent) and type II (non-estrogen-dependent) according to the pathogenesis [2]; In 2014, the World Health Organization proposed that UCEC can be classified into serous carcinoma, clear cell carcinoma, and endometrioid carcinoma based on histomorphological characteristics. However, the above methods have limitations. For example, some patients with type I endometrial cancer do not have any endocrine and metabolic disorders. While some patients with type II endometrial cancer are secondary to proliferative endometrium. In addition, the WHO classification also includes rare types of endometrial cancer, such as transitional cell carcinoma, small cell carcinoma and undifferentiated carcinoma. These types are not exactly classified as type I UCEC or type II UCEC. The inconsistency of classification methods has caused many problems in accurately predicting the prognosis of patients and guiding patients to accurate treatment solutions.

As far as the treatment options for UCEC are concerned, it is now generally accepted that surgical treatment is the mainstay, followed by comprehensive treatment such as radiotherapy, chemotherapy, targeted therapy, and immunotherapy [3–9]. For some patients who suffered from tumor progression after standard treatment, targeted therapy, and immunotherapy may be a promising rescue method.

Tumor Mutation Burden (TMB) refers to the total number of substitution and insertion/deletion mutations per megabase in the coding region of the exon of the evaluated gene in the tumor cell genome. The higher the level of TMB, the more neoantigens produced by somatic mutations, and the more easily the tumor tissue was recognized by the immune system [10,11]. Many reports have pointed out that TMB can be used as a predictor of response to immune checkpoint inhibitors [12]. In addition, the level of TMB has shown a good prognostic value in a variety of tumors (such as non-small cell lung cancer, colon cancer, liver cancer, etc.) [13–15]. Recently, the role of TMB combined with immune infiltration in endometrial cancer was studied [16]. Micro ribonucleic acid (miRNA) is a type of non-coding RNA fragment that has the function of regulating the expression of post-transcribed genes. MiRNA is a highly conserved, endogenous, non-coding single-stranded small-molecule RNA. It plays an important role in tumorigenesis and development [17–20]. In recent years, many researchers have focused on the relationship between miRNA and UCEC. It has been reported that the abnormal expression of miR-200a, miR-200 c, and miR-155 can affect the cell proliferation of UCEC [21]. MiR-195 can target SOX4 to inhibit the migration of UCEC cells [22]. In addition, miRNA can also be used as a predictor of prognosis in patients with endometrial cancer [23]. Because the calculation of TMB requires obtaining DNA files of tumor tissues, for those patients who have not undergone surgical treatment or have not undergone tissue genetic testing, it is more difficult to use TMB to predict the response to immune checkpoint inhibitors. Some researchers have proposed to use the expression of plasma miRNA to reflect the TMB level of tumor tissues [24].

The aim of our study is to establish a new molecular typing based on TMB level and identify relevant miRNAs that have the value of diagnosis, prognosis, and guidance for adjuvant therapy. Our goal is to provide valuable information for adjuvant therapy.

## Material and methods

### Data processing

With the help of the TCGAbiolinks package in the R software, we successfully obtained the mutation annotation files for UCEC [25]. In order to obtain more reliable somatic mutation results, ‘VarScan2’ software was used to identify tumor cell mutations. The ‘maftools’ package was used to read and visually analyze the somatic variants of each sample [26]. After referring to the existing literature, we defined TMB≥10 mutations per MB as a high TMB level, and conversely, defined TMB<10 mutations per MB as a low TMB level [27]. In addition, 38 MB was used as an estimate of exon size. The mature miRNA expression profile of UCEC patients was obtained through the UCSC Xena database (https://xena.ucsc.edu/public, dataset ID: TCGA.LUAD.sampleMap/miRNA_HiSeq_gene). For the obtained miRNA expression files, we further performed log2 converted reads per million (log2 (RPM + 1)) processing. UCEC samples with both mature miRNA expression files and mutation annotation files were included for further study. With the help of R software, we randomly divided patients into train set and the whole patients as the test set. The basic requirements of grouping include two aspects. First of all, we control the ratio of the number of people in train set to the number of people in test set to about 6:4. Then, from the perspective of the distribution of patients in various clinical features, the patient composition in the train set and the patient composition in the test set should be similar.

### Screening of differentially expressed miRNAs

First, in order to obtain more commonly expressed miRNAs, we deleted miRNAs that were not expressed by more than 10% of the samples in the train set. ‘Limma’ package was used to analyze the differentially expressed miRNAs between the high TMB level group and the low TMB level group [28]. When the difference met a joint satisfaction of the P value adjusted by false discovery rate (FDR) < 0.05 and |log2fold changes (FC)| >0.5, it was regarded to be statistically significant. Furthermore, bidirectional hierarchical cluster analysis was used to analyze the expression of differential miRNAs and displayed in the form of heat maps.

### Principal component analysis (PCA) before and after least absolute shrinkage and selection operator (LASSO)

In order to obtain diagnostic miRNAs and prevent overfitting results, the LASSO logistic regression model analysis was used to determine the most suitable miRNAs to reflect the TMB level of UCEC patients in train set. The implementation of the LASSO method was achieved through the ‘glmnet’ package [29]. The LASSO regression is an approach for variable selection in fitting high-dimensional generalized linear model. Then, to make it clear that the miRNAs we selected can well distinguish samples with different TMB levels, PCA was performed, respectively, on the expression profiles of all miRNAs before LASSO treatment and the expression profiles of miRNAs with diagnostic value after LASSO treatment.

### Construction of the miRNA-based diagnostic model

We constructed a miRNA-based diagnostic model for predicting TMB level using the expression of diagnostic miRNAs and the corresponding regression coefficients obtained from LASSO regression.

The diagnostic model was calculated using the following formula: β1 × miRNA1 expression + β2 × miRNA2 expression + … + βn × miRNAn expression, where β corresponded to the corresponding regression coefficient.

In order to evaluate the value of the classifier we constructed to predict the TMB level of UCEC patients, the ROC curves were performed in the train set, test set, and entire set. Then, the objective scores are given in terms of sensitivity (Se), specificity (Sp), positive predictive value (PPV), negative predictive value (NPV), accuracy, and area under the receiver operating characteristic curve (AUC) through the ‘pROC’ package in the R software [30].

### The correlation between the miRNA-based diagnostic model and the expression of five immune checkpoints, the infiltrating levels of several types of immune cell

The mRNA expression data of UCEC patients were downloaded from the TCGA Data Portal (https://tcga-data.nci.nih.gov/tcga/; accessed 15 May 2020). The ‘Limma’ package was used for differential analysis of gene expression. The ‘CIBERSORT’ tool was used to quantify immune cell fractions [31]. P < 0.05 was settled as the threshold, only those that met the CIBERSORT threshold were deemed as qualified for the subsequent analysis. We then analyzed the correlation between the miRNA-based diagnostic model and the current mainstream immune checkpoint regulators expression. Further, the correlation between this model and the infiltrating levels of several types of immune cell in tumor tissues has also been studied.

### Functional enrichment analysis

Through DIANA-mirPath web-server, we obtained mRNAs that interact with selected miRNAs [32]. In addition, we also performed the KEGG pathway enrichment analysis and gene ontology (GO) enrichment analysis on selected miRNAs. We set p < 0.05 as the threshold, and only the KEGG pathways, and GO terms that met the threshold were considered to be significantly enriched.

### Identification prognostic related miRNAs and establishment of a miRNA-based model for predicting the prognosis of UCEC patients

The ‘Limma’ package was again used to discover the differentially expressed miRNAs at different TMB levels in the entire TCGA set. We defined the obtained miRNAs differentially expressed between the high TMB level group and the low TMB level group as targeted miRNAs.

And we obtained data from 497 patients randomly selected from 300 patients as the validation cohort by using the ‘caret’ package in R software. First, univariate Cox proportional hazard regression analysis was used to identify survival-related miRNAs in the train set when the p-value was <0.05. Then, the least absolute shrinkage and selection operator (LASSO) Cox regression analysis was performed using ‘glmnet’ package. Finally, multivariate Cox regression analysis was employed to determine the key miRNAs with the most prognostic value in UCEC patients. A miRNA-based prognostic risk signature (PRS) for predicting the prognosis of UCEC patients was established according to the following formula: ExpmiRNA1*Coef1 + ExpmiRNA2*Coef2 + ExpmiRNA3 *Coef3+ … ExpmiRNAn*Coefn where Coef refers to the regression coefficient of the corresponding miRNA obtained from the Cox regression analysis.

### Evaluation of the PRS

To evaluate whether our model could be used as an independent prognostic factor, we included age, stage, histological type, grade, and PRS as independent variables. We then performed univariate Cox regression analysis and multivariate Cox regression analysis on the changes in overall survival time and overall survival outcome.

Further evaluation of the established prognostic model mainly includes two aspects. On the one hand, ROC curves of different variables in the TCGA cohort at 1 year, 3 years, and 5 years were created by the ‘survivalROC’ package [33]. Corresponding AUC values were also calculated to evaluate the accuracy of different variables for predicting the prognosis of UCEC patients.

In addition, Gene-set enrichment analysis was used to explore the mechanisms that lead to different outcomes between patients in the high-risk group and patients in the low-risk group. By means of ‘rms’ package of R software, a prognostic nomogram was also performed to visualize the relationship between individual predictors and overall survival rates in patients with UCEC.

### IPS analysis

IPS can be derived using machine learning based on four major categories of immunogenicity determining genes. The IPS is calculated based on representative cell-type gene expression z-scores, higher scores mean increased immunogenicity. The IPS scores of UCEC patients were obtained from the Cancer Immunome Atlas (TCIA) (https://tcia.at/home) [34].

### Estimate of tumor-infiltrating immune cells (TIICs)

We used the CIBERSORT tool to quantify 22 types of immunocyte fractions based on TCGA RNA-sequencing data. P < 0.05 was settled as the threshold.

### Chemotherapy response prediction

The response of chemotherapy was predicted by the public pharmacogenomics database Genomics of Drug Sensitivity in Cancer (GDSC; https://www.cancerrxgene.org). The half-maximal inhibitory concentration (IC50) was estimated and represented the response of the drug.

### Statistical analysis

Statistical analyses of all the data utilized in this article were completed by R software (version 16 3.5.1, https://www.r-project.org/). When the difference met a joint satisfaction of FDR < 0.05 and |log_2_fold changes (FC)| >0.5, it was regarded to be statistically significant. Student’s t test was used for continuous variables, while categorical variables were compared with the chi-square (χ2) test. The Wilcoxon rank-sum test was utilized to compare ranked data with two categories. The Kruskal–Wallis test was utilized for comparisons among three or more groups. The LASSO regression analysis was used to identify optimal miRNAs reflecting the TMB levels. The LASSO regression analysis and 10-fold cross validation were used to evaluate the relationship between targeted miRNAs expression and survival data to establish a prognostic model. ‘rms’ package of R software was used to create the nomogram. The ROCs were created by the ‘survivalROC’ package of R software and the AUC values were also calculated by this package. All the statistical tests were two-sided and P < 0.05 was considered to be statistically significant.

## Results

In this study, we constructed a miRNA-based prognostic signature through the TCGA database, and we hypothesized that the signature can predict the prognosis of patients with UCEC, and the goal of our study is to provide valuable information for the clinical practice of patients with UCEC. In this study, we used data from UCEC patients from the TCGA database, filtered miRNAs to establish a signature. The prognostic performance of the signature was evaluated and validated. Further, the predicted response to immunotherapy and chemotherapy was also investigated.

### Differentially expressed miRNAs between high TMB level group and low TMB level group

**Figure 1.**
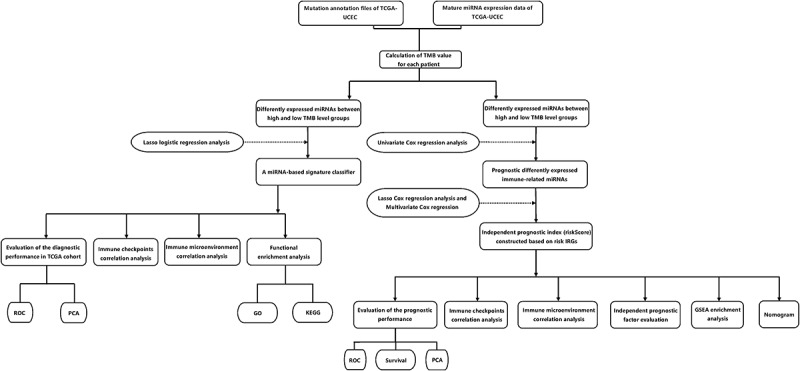
Flow Chart of this research

The total design and process of this research were presented in the flow chart (Figure 1). First, we included patients with TMB ≥ 10 mutations per MB into the high TMB level group, and the remaining patients into the low TMB level group. Then, in order to facilitate the establishment of subsequent diagnostic model and prognostic model, we randomly divided all UCEC patients into two groups (training set and test set). In the training set, 112 patients with high TMB levels and 199 patients with low TMB levels were included. There was a total of 25 significantly differentially expressed miRNAs between the two groups (including 7 up-regulated and 18 down-regulated) in the training set (Figure S1). The routine clinical characteristics of all patients included in the study were shown in Table 1. From Table 1, we found that there was no significant difference in the distribution of clinical features between the groups.

**Table 1.** Clinical factors of patients in three cohorts sets

Characteristic		Entire set	Train set	Test set	P value
Age	≤60	192(39.34%)	113(38.57%)	79(40.51%)	0.7365
	>60	296(60.66%)	180(61.43%)	116(59.49%)	
Stage	Stage I & Stage II	355(72.75%)	209(71.33%)	146(74.87%)	0.4493
	Stage III Stage IV	133(27.25%)	84(28.67%)	49(25.13%)	
Histological	endometrial	369(75.61%)	216(73.72%)	153(78.46%)	0.277
type	Mixed and serous	119(24.39%)	77(26.28%)	42(21.54%)	
Grade	G1 & G2	88(18.03%)	56(19.11%)	32(16.41%)	0.5219
	G3 & G4	400(81.97%)	237(80.89%)	163(83.59%)	
Fustat	Alive	407(83.4%)	243(82.94%)	164(84.1%)	0.8295
	Dead	81(16.6%)	50(17.06%)	31(15.9%)

### Identification of miRNAs that could reflect TMB levels

**Figure 2.**
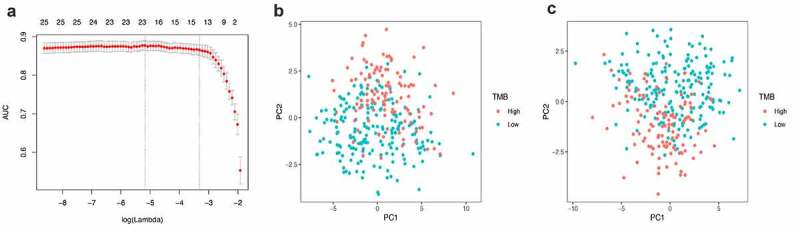
Lasso regression analysis and principal component analysis. (a) 10-fold cross-validation in the Lasso regression analysis. (b) principal component analysis before and after (c) Lasso variable reduction

The LASSO logistic regression method was used to further determine the miRNAs that best reflect the TMB level. Finally, 21 miRNAs were identified as diagnostic miRNAs for predicting TMB levels (Figure 2(a)). Figure 2(b,c) respectively shows that PCA is constructed based on all differential miRNAs and the PCA constructed based on 21 diagnostic miRNAs, which suggests that the diagnostic miRNAs determined by the LASSO logistic regression method can better distinguish patients with high TMB levels from patients with low TMB levels.

### The LASSO logistic regression classifier

After obtaining miRNAs that could well reflect the TMB levels, we used these miRNAs to construct a classifier to better judge the TMB levels of patients. Table 2 shows the coefficient of 21 target miRNAs derived from the LASSO cox regression. The diagnostic model was calculated using the following formula: index = hsa-miR-146a-5p * 0.091672 + hsa-miR-708-5p * (−0.01454) + hsa-miR-4746-5p * 0.647021 + hsa-miR-452-5p * 0.057283 + hsa-miR-452-3p * (−0.26965) + hsa-miR-224-5p * 0.018647 + hsa-miR-375-3p * 0.11641 + hsa-miR-30a-5p * 0.328458 + hsa-miR-598-3p * 0.022044 + hsa-miR-335-3p * (−0.49775) + hsa-miR-30 c-5p * (−0.63721) + hsa-miR-101-5p * 0.021696 + hsa-miR-210-3p * 0.571997 + hsa-miR-676-3p * (−0.53052) + hsa-miR-130a-3p * 0.021182 + hsa-miR-1266-5p * 0.346479 + hsa-miR-1271-5p * (−0.10099) + hsa-miR-130a-5p * (−0.09051) + hsa-miR-203b-3p * (−0.06494) + hsa-miR-3074-5p * 0.432032 + hsa-miR-30d-5p * (−0.40024).

**Table 2.** The coefficient of target miRNAs derived from the LASSO Cox regression

Gene	Coefficient
hsa-miR-146a-5p	0.091672
hsa-miR-708-5p	−0.01454
hsa-miR-4746-5p	0.647021
hsa-miR-452-5p	0.057283
hsa-miR-452-3p	−0.26965
hsa-miR-224-5p	0.018647
hsa-miR-375-3p	0.11641
hsa-miR-30a-5p	0.328458
hsa-miR-598-3p	0.022044
hsa-miR-335-3p	−0.49775
hsa-miR-30 c-5p	−0.63721
hsa-miR-101-5p	0.021696
hsa-miR-210-3p	0.571997
hsa-miR-676-3p	−0.53052
hsa-miR-130a-3p	0.021182
hsa-miR-1266-5p	0.346479
hsa-miR-1271-5p	−0.10099
hsa-miR-130a-5p	−0.09051
hsa-miR-203b-3p	−0.06494
hsa-miR-3074-5p	0.432032
hsa-miR-30d-5p	−0.40024

**Figure 3.**
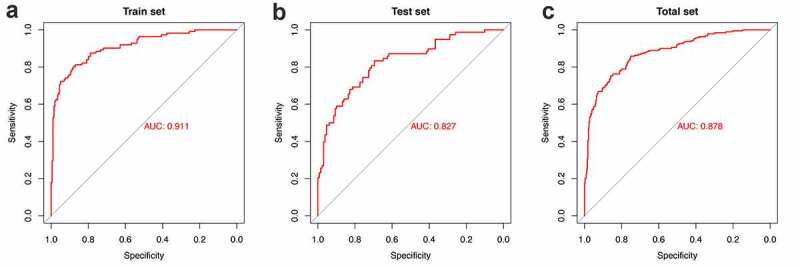
The receiver operating characteristic curves for miRNA-based diagnostic model. (a-c) The ROC curves in the train set, test set and entire set, respectively

Figure 3 shows ROC curves for 21-miRNA-based diagnostic model in the training set, test set, and entire set, respectively. The AUC value was 0.911 in the training set, 0.827 in the test set and 0.878 in the entire set. Table 3 shows the SE, SP, PPV, NPV and Accuracy values of 21-miRNA-based classifiers of TMB in UCEC patients. From which, we can find that the classifier we built shows excellent diagnostic value whether it is in the training set, test set, or entire set.

**Table 3.** Performance of 21-miRNA-based classifiers of TMB in UCEC patients

ID	SE	SP	PPV	NPV	Accuracy	AUC
Train set	0.7768	0.8945	0.8056	0.8768	0.8521	0.9112
Test set	0.6282	0.8516	0.7206	0.7899	0.767	0.8272
Entire set	0.7158	0.8777	0.7727	0.8416	0.8182	0.8783

### The correlation between the miRNA-based diagnostic model and the expression of five immune checkpoints, the infiltrating levels of several types of immune cell

**Figure 4.**
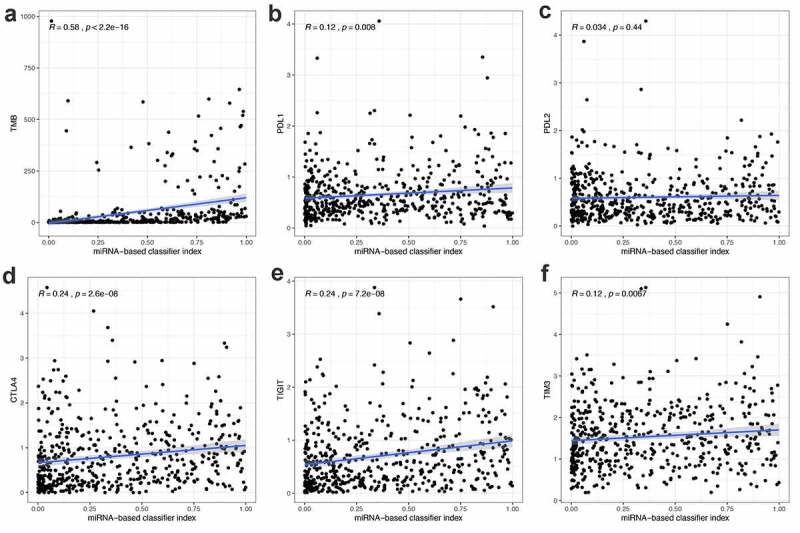
The correlation between miRNA-based diagnostic model and the expression of TMB, PDL1, PDL2, CTLA4, TIGIT and TIM3. (a-f) The miRNA-based diagnostic model shows positive correlation with (a) TMB, (b) PDL1 expression, (c) PDL2 expression, (d) CTLA4 expression, (e) TIGIT and (f) TIM3 expression

To explore whether the 21-miRNA-based diagnostic model could accurately guide immunotherapy, we studied the relationship between the classifier and the expression level of immune checkpoints. The relationship between the classifier and the infiltration of immune cells was also studied. From Figure 4, we could find that the 21-miRNA-based diagnostic model shows positive correlation with TMB (*R* = 0.58, *p* < 2.2e−1), PDL1 expression (*R* = 0.12, *p* = 0.008), CTLA4 expression (*R* = 0.24, *p* = 2.6e−08), TIGIT expression (*R* = 0.24, *p* = 7.2e−08), and TIM3 expression (*R* = 0.12, *p* = 0.0067). PDL2 expression seemed to have no correlation with the diagnostic model (*R* = 0.12, *p* = 0.0067) (Figure 4(c)). Figure S2 shows the correlation between 21-miRNA-based diagnostic model and infiltrating levels of several types of immune cell. We found that the 21-miRNA-based diagnostic model has a significant correlation with the infiltration of six kinds of immune cells. Among them, it shows a positive correlation with the infiltration of T cells CD4 memory activated (Cor = 0.320, *p* = 3.76e−08), and the infiltration of T cells CD8 (Cor = 0.179, *p* = 0.002)). While it shows a negative correlation with the infiltration of mast cells resting (Cor = −0.134, *p* = 0.024)), the infiltration of T cells CD4 memory resting (Cor = −0.190, *p* = 0.001), the infiltration of B cells memory (Cor = −0.130, *p* = 0.029), and the infiltration of dendritic cells activated (Cor = −0.129, *p* = 0.030).

### Functional enrichment analysis

Functional enrichment analysis, including KEGG pathway enrichment analysis and GO enrichment analysis, was used to explore the potential functions of the 21 diagnostic miRNAs. From Figure S3A, we can find that most of these miRNAs are related to pathways related to cancer (including PI3K-Akt signaling pathway, MAPK signaling pathway, Rap1 signaling pathway, Ras signaling pathway, cAMP signaling pathway, and so on). From Figure S3B, we unexpectedly found that these miRNAs are enriched in many immune-related biological events. The three most enriched three biological processes are immune system processes, innate immune processes and neurotrophic TRK receptor signaling pathways. These results also further illustrate that the 21-miRNA-based diagnostic model we have established can reflect the immune microenvironment to a certain extent. Furthermore, the results of molecular function enrichment analysis and cellular component enrichment analysis are shown in Figure S3C-D.

### Establishment of an independent prognostic model based on miRNAs in train set

Firstly, we divide all UCEC into high TMB level group and low TMB level group according to the value of TMB. Then, ‘Limma’ package was used to analyze the differences in the expression of miRNAs between the two groups and differential miRNAs related to the level of TMB were successfully identified (Including 61 down-regulated miRNAs and 10 up-regulated miRNAs) (Figure S4A). Second, in order to facilitate the training and verification of the prognostic model, 300 patients as training set were randomly assigned from the 497 patients (entire set).

In the training set, we carried out a univariate Cox regression analysis to identify miRNAs related to the UCEC patient’s overall survival (OS). In the end, a total of 16 miRNAs were considered to be prognostic miRNAs. Then, LASSO regression analysis was used to prevent overfitting (Figure S4B-C). After that, the multivariate Cox regression analysis was performed on the training set to filter the key miRNAs. Finally, three miRNAs were identified. Among the three miRNAs (Figure S4D), hsa-miR-139-5p and hsa-miR-1301-3p were considered as predictors of poor prognosis. The higher the expression of hsa-miR-139-5p and hsa-miR-1301-3p, the worse the prognosis of patients. While hsa-miR-146a-5p was a protective factor.

Based on the results of multivariate Cox regression analysis, we obtained the risk coefficient of these miRNAs and constructed a prognostic risk signature (PRS) to predict the prognosis of patients with UCEC. These three prognostic related miRNAs related PI formula was as follows: (hsa-miR-146a-5p expression) * (−0.287626402898921) + (hsa-miR-139-5p expression) * (0.207299937064514) + (hsa-miR-1301-3p expression) * (0.237447660931411). Therefore, each patient can be assigned a risk score. We set the median of the risk scores of all patients in the training set as a threshold. Then, we divide patients with risk scores greater than or equal to the threshold into the high-risk group and others into the low-risk group.

### Evaluation of the prognostic model in the train set and entire set

**Figure 5.**
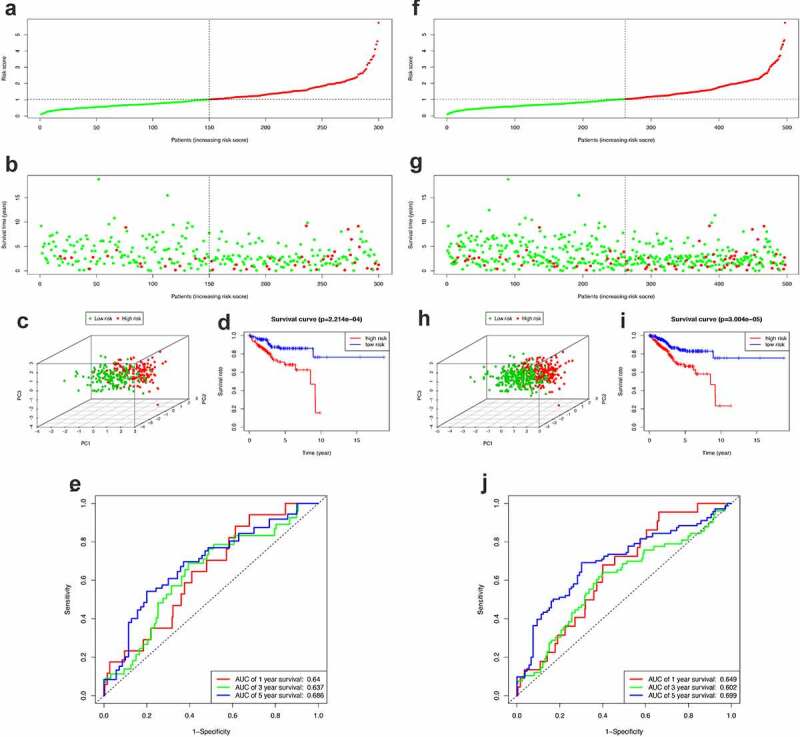
Construction of the PRS. (a-c) The distribution of Risk score, survival status and PCA in training set. (d) Kaplan-Meier survival curves of over survival between high-risk and low-risk patients in training set. (e) 1-year, 3-year, and 5-year ROC curve of the predictive power of the PRS in training set. (F-H & I-J) The similar analyses which were conducted in the entire set

After obtaining the UCEC patients’ prognostic model, we took a series of measures to evaluate this model. Firstly, the survival status of patients in the high-risk group and the low-risk group is shown in Figure 5. Figure 5(a,f) show the result of risk classification of patients in the training set and in the entire set according to PRS, respectively. From Figure 5(b,g) we found that no matter in the training set or in the entire set as the risk score increases, the number of dead patients increases. Then, PCA based on the expression of key miRNAs was implemented to evaluate the discrimination of patients with different survival conditions (Figure 5(c,h). The PCA results suggest that whether in the training set or the entire set, high-risk patients and low-risk patients could be effectively distinguished based on PRS. Then, we created Kaplan–Meier curves based on the log-rank test to visualize the prognostic value of our established prognostic model in the training set and in the entire set. From Figure 5(d,i), we found that whether in the training set or the entire set, patients with high riskScore have a poor prognosis. The time-dependent ROC curves of the PRS in predicting the prognosis of patients in the training set and the entire set were presented in Figure 5(e,j). In the training set, AUC of the prognostic model at 1 year, 3 years, and 5 years were 0.64, 0.637, and 0.686, respectively. In the entire set, the AUC of the prognostic model at 1 year, 3 years, and 5 years were 0.649, 0.602, and 0.699, respectively.

To further evaluate whether this model could be used as an independent prognostic factor, we added some key clinical characteristics including age, stage, histological type, grade, and PRS as independent variables. By means of univariate and multivariate Cox regression analysis, PRS remained significant (P ≤ 0.001, Table 4) in both the training set and the entire set. At the same time, the results of multivariate Cox regression analysis showed that stage could also be used as independent prognostic indicators (P < 0.05) in both the training set and the entire set.

**Table 4.** Univariate and multivariate Cox regression analyses of the prognosis-related factors

Variables	Univariate analysis			Multivariate analysis		
	HR	95%CI	P-value	HR	95%CI	P-value
**Train sets**						
Age	2.066	1.055–4.045	0.034	1.827	0.891–3.749	0.100
Stage	3.803	2.164–6.682	<0.001	3.232	1.767–5.914	<0.001
Histological type	2.865	1.637–5.014	<0.001	1.112	0.571–2.166	0.754
Grade	3.226	1.566–6.644	0.001	1.523	0.664–3.493	0.320
RiskScore	1.855	1.447–2.379	<0.001	1.621	1.230–2.137	<0.001
**Test sets**						
Age	1.981	1.204–3.260	0.007	1.803	1.068–3.041	0.027
Stage	4.154	2.670–6.462	<0.001	3.507	2.189–5.617	<0.001
Histological type	2.928	1.886–4.547	<0.001	1.012	0.597–1.717	0.964
Grade	3.950	2.179–7.159	<0.001	2.162	1.119–4.179	0.022
RiskScore	1.721	1.400–2.115	<0.001	1.491	1.184–1.878	0.001

HR: hazard ratio; CI: confidence interval

**Figure 6.**
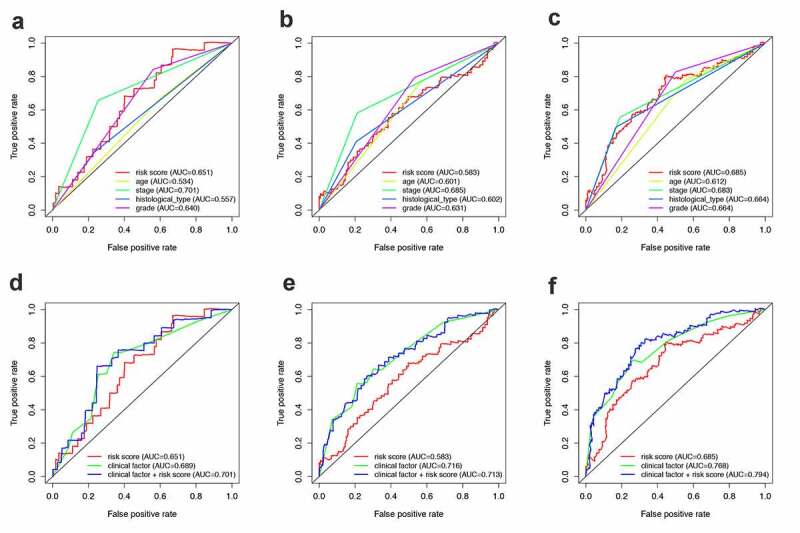
The predictive power of the PRS and other clinical characteristics. (a-c) 1-, 3- and 5-year ROC of PRS and the other clinical characteristics. (d-e) 1-, 3- and 5-year ROC of the combining of PRS and the existing clinical factors

In addition, the ROC curves of multiple prognostic indicators were created to visualize the prognostic value of our established prognostic model in the entire set (Figure 6). Figure 6(a-c), respectively, shows the 1-, 3- and 5-year ROC of RPS and the other clinical characteristics. While Figure 6(d-f), respectively, shows the 1-, 3- and 5-year ROC of combining of IRPS and the existing clinical factors. It was worth mentioning that the predictive ability of the prognostic model was better than clinical characteristics.

### Nomogram development and validation for prognostic risk prediction

**Figure 7.**
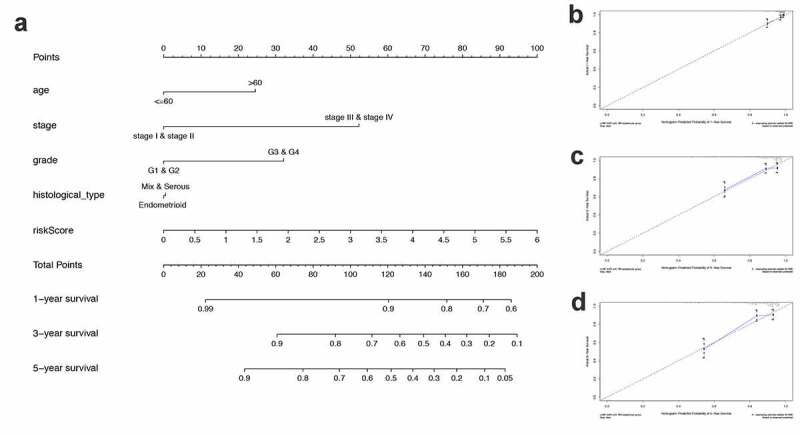
Construction and validation of a nomogram. (a) A nomogram to predict the probability of 1-, 3- and 5-year OS of UCEC patients. (b-d) Calibration curves of the nomogram to predict the probability of OS at 1, 3 and 5 years

Finally, to better predict the 1-year OS, 3-year OS, and 5-year OS of UCEC patients, we constructed a nomogram based on PRS and clinical features (Figure 7(a)). Figure 7(b-d) show the calibration curves of the nomogram for the probability of OS at 1, 3, and 5 years.

### Gene-set enrichment analysis

To further explore the possible mechanisms that caused different outcomes in the high-risk group and the low-risk group, Gene-set enrichment analysis (GSEA) was performed. Figure S5A shows enriched pathways in the high-risk group, while Figure S5B shows enriched pathways in the low-risk group. The results of GSEA suggested that most of the differentially expressed genes in the low-risk group were genes related to immune pathways.

### The correlation between PRS and clinical features, immune checkpoints, and immune cell infiltration

**Figure 8.**
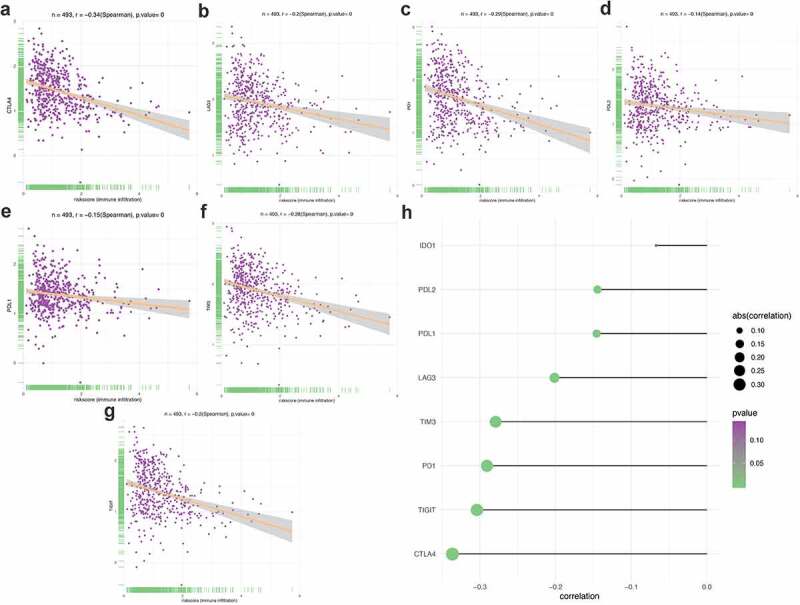
Correlation between PRS and the expression of immune checkpoint regulators. (a-g) The association between PRS and the expression of each immune checkpoint regulators. (h) The landscape of the association between PRS and some immune checkpoint regulators

**Figure 9.**
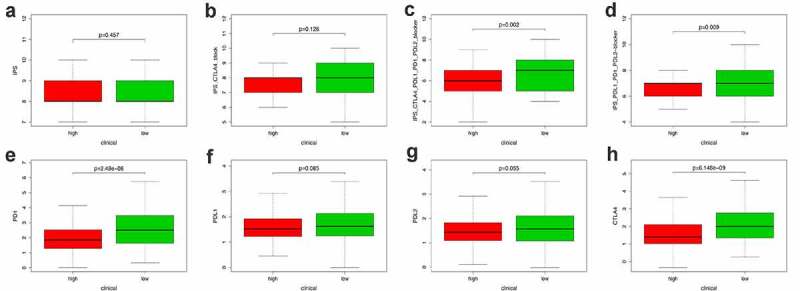
Correlation between PRS and immune checkpoint regulators. (a-d) The association between PRS and IPS in UCEC patients. (e-h) The association between PRS and the expression of checkpoints in UCEC patients

**Figure 10.**
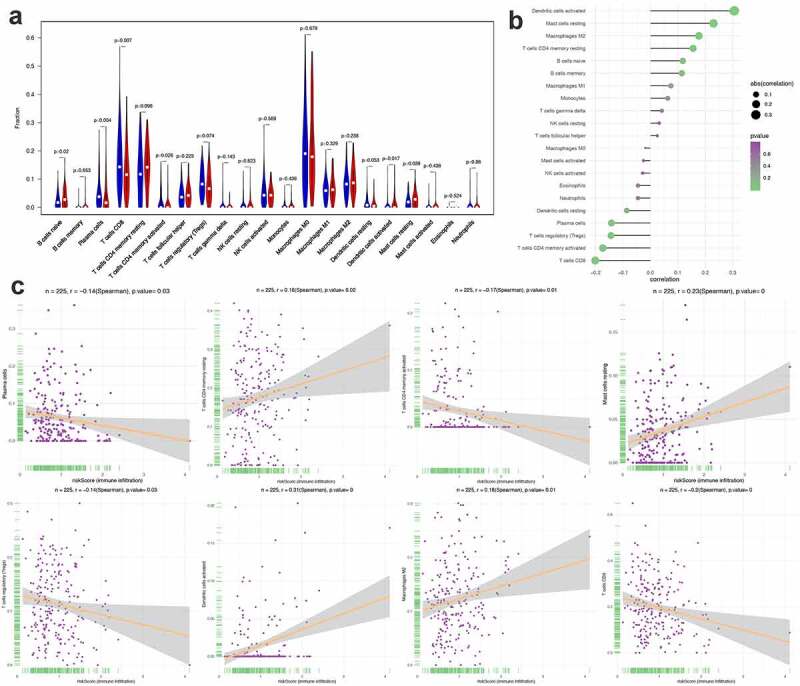
Correlation between PRS and immune cell infiltration. (a) The landscape of immune cell infiltration in low-risk and high-risk groups. A blue violin represents the low-risk group. A red violin represents the high-risk group. (b) The association between PRS and immune cell infiltration. (c) The association between PRS and each type of immune cell

Apart from the mentioned conventional methods of evaluating prognostic ability, we also explored the correlation between PRS and clinical characteristics (Figure S6), immune checkpoint regulators (Figures 8 & 9) and immune cell infiltration (Figure 10). PRS was positively associated with tumor stage, grade, and histology. Patients with early stage and low-grade tumors were more likely to be assigned a lower risk score, compared with other histological-type patients with endometrial tumor (Figure S1). When it turns to the immune aspect, PRS showed negative association with several immune checkpoint regulators (Figure 8), especially the CTLA4 expression (Figure 8(a)), the TIGIT expression (Figure 8(g)), and the PD1 expression (Figure 8(c)). To further explore whether PRS can be used to guide immunotherapy, we compared the expression of immune checkpoints and immunophenoscores (IPS) between two groups (Figure 9). The results showed that PD-1 and CTLA4 were highly expressed in patients in the low-risk group (Figure 9(e,h). In addition, similar results were obtained in terms of immunogenicity. The IPS_CTLA4_PD-L1_PD-1_PD-L2 and IPS_PD-L1_PD1_PD-L2 scores were higher in patients in the low-risk group (Figure 9(c,d)). Emerging evidence has confirmed the effect of PD-1 inhibitor in treating UCEC [35,36]; however, the therapy response in different patients was different. In this study, patients in the low-risk group were more likely to benefit from several immune checkpoint inhibitors, especially the PD-1 inhibitors.

Furthermore, we found that there are significant differences in the infiltration of multiple immune cells between the high-risk group and the low-risk group. Among them, the density of B cell naive, dendritic cells activated and Mast cells resting in the high-risk group was significantly higher than that in the low-risk group. In contrast, in the low-risk group, the density of Plasma cells, T cells CD8+ and T cells CD4 memory activated was significantly higher than that of the high-risk group (Figure 10(a)). In this study, we also investigated the correlation between PRS and the infiltration of immune cells, the results showed that PRS was positively associated with the infiltration of immune cells like dendritic cell activated, mast cell resting, macrophages M2, T cell CD4 memory resting, B cell native and B cell memories. At the same time, PRS showed negative correlation with dendritic cell resting, plasma cells, T cell regulatory, T cells CD4 memory activated and T cells CD8 (Figure 10(b-c)). These results imply that immunological recognition activities in patients with high PRS scores are fairly strong.

**Figure 11.**
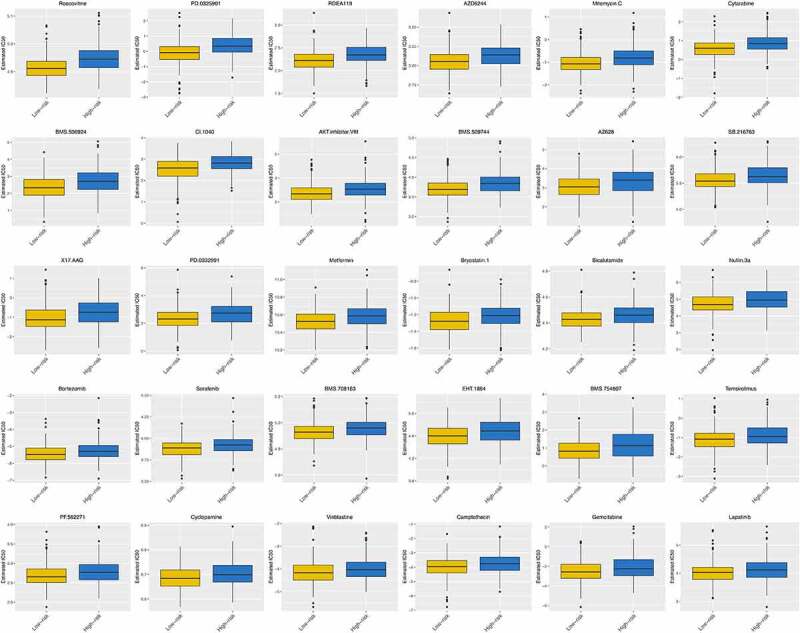
Different response to chemotherapeutic regimens in the high-risk and low-risk groups (P < 0.05)

Figure 11 shows the differences in the response to some commonly used chemotherapy regimens between the high-risk group and the low-risk group. From which we can find that patients in the low-risk group have shown good therapeutic effects on some common anti-tumor drugs, such as roscovitine, cytarabine, bicalutamide, cyclopamine, gemcitabine, and so on.

## Discussion

As the sixth most common gynecological malignant tumor in the world, endometrial cancer has an increasing morbidity and mortality year after year. With the in-depth study of the molecular mechanism of UCEC, immunotherapy of UCEC have gradually become the focus of research. For some patients not suitable for conventional treatment, immunotherapy is the alternative. However, these patients showed significant heterogeneity in their response to immunotherapy [37]. In addition, existing prognostic stratification systems are mainly based on clinical and pathological parameters with a low accuracy [38]. Therefore, more precise biomarkers that can reflect the tumor microenvironment and predict the effectiveness of immunotherapy are essential to improve perioperative risk assessment and guide treatment decisions.

Our study identified several miRNAs in UCEC patients that are related to the tumor microenvironment. In order to obtain a high-accuracy miRNA-based diagnostic model, a series of scientific and rigorous methods were carried out to construct the model. We then verified the constructed model in many aspects. Firstly, we collated the gene expression data and corresponding clinical data of UCEC patients, and the TMB data of each patient using the methods previously reported [39]. Then, we divided UCEC patients into high TMB level group and low TMB level group based on TMB. The differentially expressed miRNAs between the two groups were used to further explore TMB-related miRNAs. We divide UCEC patients into training set and test set according to the distribution of clinical characteristics (age, stage, histological type, and grade). After Lasso regression analysis and principal component analysis, we successfully established a 21-miRNA-based classifier of TMB in UCEC patients. The AUC of the ROC curves further determines the accuracy of this diagnostic model (0.911 on the training set, 0.827 on the test set and 0.878 on the entire set). In order to more intuitively judge that whether the miRNA-based classifier, we obtained can well reflect the tumor microenvironment, the correlation between the miRNA-based diagnostic model and the expression of five immune checkpoints and the infiltrating levels of several types of immune cells were revealed. What we found interesting is that this miRNA-based diagnostic model has a significant positive correlation with TMB, PD-L1, CTLA4, TIGIT, TIM3. In addition, the miRNA-based diagnostic model shows a negative correlation with the infiltrating levels of mast cells resting, T cells CD4 memory resting, B cells memory, and dendritic cells activated. At the same time, the miRNA-based diagnostic model shows a positive correlation with the infiltrating levels of T cells CD4 and T cells CD8. We then performed functional enrichment analysis on 21 miRNAs and enriched many immune and cancer-related pathways. The above results indicate that the 21-miRNA-based diagnostic model we established can reflect the TMB level and immune activity of UCEC patients very well. However, the relationship between TMB and the efficacy of immunosuppressants have been confirmed. Emerging evidence have found that miRNA may be involved in regulating the immune activity of tumors [40–42], we believe that the diagnostic model we have established has high feasibility.

In addition, we further explored the relationship between immune-related miRNA and the survival of UCEC patients, and established a PRS to predict the prognosis of UCEC patients. The results of the GESA analysis of UCEC based on PRS are also very interesting. We found that the poor prognosis of patients in the high-risk group was related to some cancer signaling pathways. On the other hand, the better prognosis of patients in the low-risk group might be caused by changes in immune-related pathways.

Apart from reflecting the prognosis-related information of patients, PRS can also predict the response to immune therapy and chemotherapy. Choosing suitable therapy and specific drugs is a great concern for every patient, due to the complicated and special tumor microenvironment of each patient, the response to immune therapy or chemotherapy is distinct to some degree. In this research, we found that PRS was negatively corrected with the expression of several immune checkpoints, especially the PD-1 expression. The existing researches have confirmed the effect of PD-1 inhibitor in treating UCEC; however, the therapy response in different patients was distinct [43]. At the same time, therapy response predict models are limited. In our research, we found that patients in high-risk groups may not benefit from PD-1 inhibitor therapy or even other commonly used immune therapy based on immune checkpoint inhibitors. Other treatment regimens should be applied. Meanwhile, for patients in the low-risk group, immune checkpoint therapy might be a good choice. In further exploration of the immune microenvironment, we found that PRS can also reflect the immune cell infiltration in the tumor of UCEC patients. One interesting thing we found is that the density of activated dendritic cells, macrophages M2, resting Mast Cells and resting CD4 memory T cells is positively correlated with *riskScore*. In contrast, patients with a higher density of Plasma cells activated CD4 memory T cells, CD8 + T cells and Tregs cells have lower *riskScore* value. Among them, CD8 + T cells have strong tumor-killing properties and have been proved to have the value of predicting benign prognosis in many solid cancers [44]. Tregs cells, a subtype of CD4 + T cells, have different prognostic effects in different tumors. According to reports, in bladder cancer and head and neck cancer, tregs cells are associated with a benign prognosis, while in kidney cancer and cervical cancer, they play a completely opposite role [45–47]. Macrophage M2 has a clear anti-inflammatory effect. Therefore, the macrophage M2 can promote the immune tolerance of tumors, thus leading to poor efficacy of immunotherapy [48]. Dendritic cells are the key antigen-presenting cells that determine the activation and differentiation of T cells. It has been reported that activated dendritic cells are associated with a poor prognosis of colorectal cancer [49]. Evidence of the prognostic effect of these immune cell infiltration conditions on other tumors shows that PRS we constructed can accurately reflect the immune microenvironment of UCEC patients. What’s more, patients in the low-risk group have higher IPS than those in the high-risk group. IPS has been approved as an in vitro diagnostic test indicator for colorectal cancer immunotherapy, and is expected to become a reliable indicator of immune efficacy [50]. Therefore, PRS we constructed can not only reflect the prognosis of UCEC but also predict the immune microenvironment of the tumor and provide guidance for the choice of immunotherapy. When it turns to the chemotherapy, patients in the low-risk groups might be sensitive to several chemotherapeutic regimens listed in the Figure S5.

With the help of RPS, clinicians can obtain a general impression of patients’ response to immune therapy or chemotherapy, which can assist clinicians in choosing most suitable drugs for their patients and avoiding invalid treatment.

However, our research still has many limitations. First, the miRNA-based TMB diagnostic model and the prognostic model are both based on data from public databases and have not been verified by follow-up trials. What is more, it has been reported that the prognosis of endometrial cancer is directly related to some clinical factors, such as the time of diagnosis, depth of myometrial invasion, cervical involvement, tumor size, lymphovascular space invasion (LVSI), and lymph node status [2,51]. However, due to incomplete clinical data, we failed to incorporate all the reported risk factors into consideration for comprehensive analysis. In addition, the molecular mechanism of how these immune-related miRNAs regulate the immune activity of tumors and affect the occurrence and development of tumors has not yet been explored. Further experiments in vivo and in vitro are needed.

## Conclusion

A 21-miRNA-based diagnostic model which could accurately predict the TMB level of UCEC patients was successfully established, it can also predict the prognosis of UCEC patients and the response to chemotherapy and immunotherapy, thus providing valuable information on the choice of treatment regimen. A prognosis-related model based on three immune-related miRNAs was also successfully established to predict the prognosis of patients with UCEC. Our research may provide new sights in UCEC patients’ prognosis and treatment management.

## Supplementary Material

Supplemental Material

## Data Availability

The datasets in this study are available from TCGA (https://portal.gdc.cancer.gov) .
