# Supplementary figures and images for: MiRNA based tumor mutation burden diagnostic and prognostic prediction models for endometrial cancer
Source: Bioengineered. 2021 Jul 12;12(1):3603–20. doi: 10.1080/21655979.2021.1947940 (PMC8806700; doi:10.1080/21655979.2021.1947940)

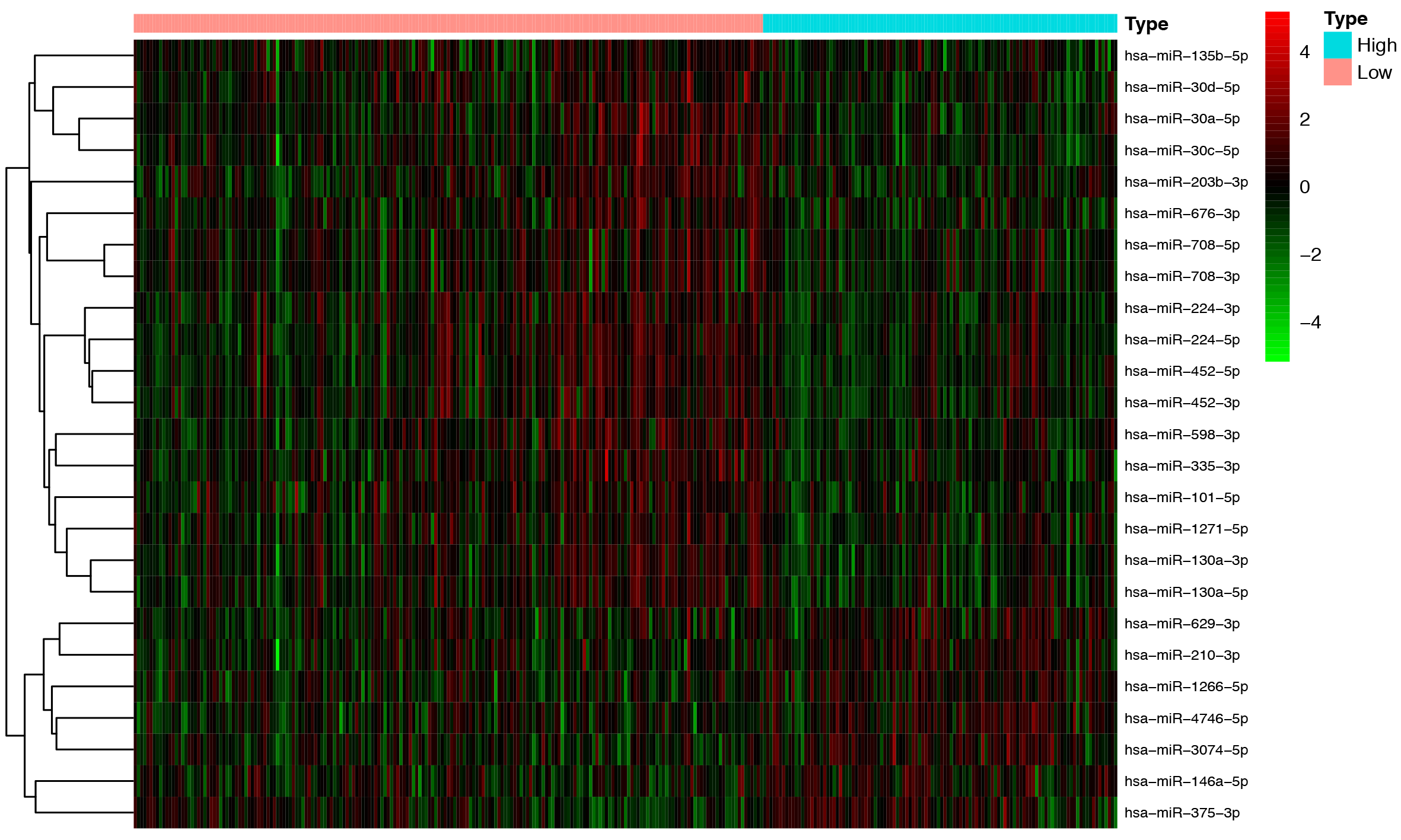

Supplement: Supplemental Material [file KBIE_A_1947940_SM6951.zip › supplementary/FIG S1.tif]

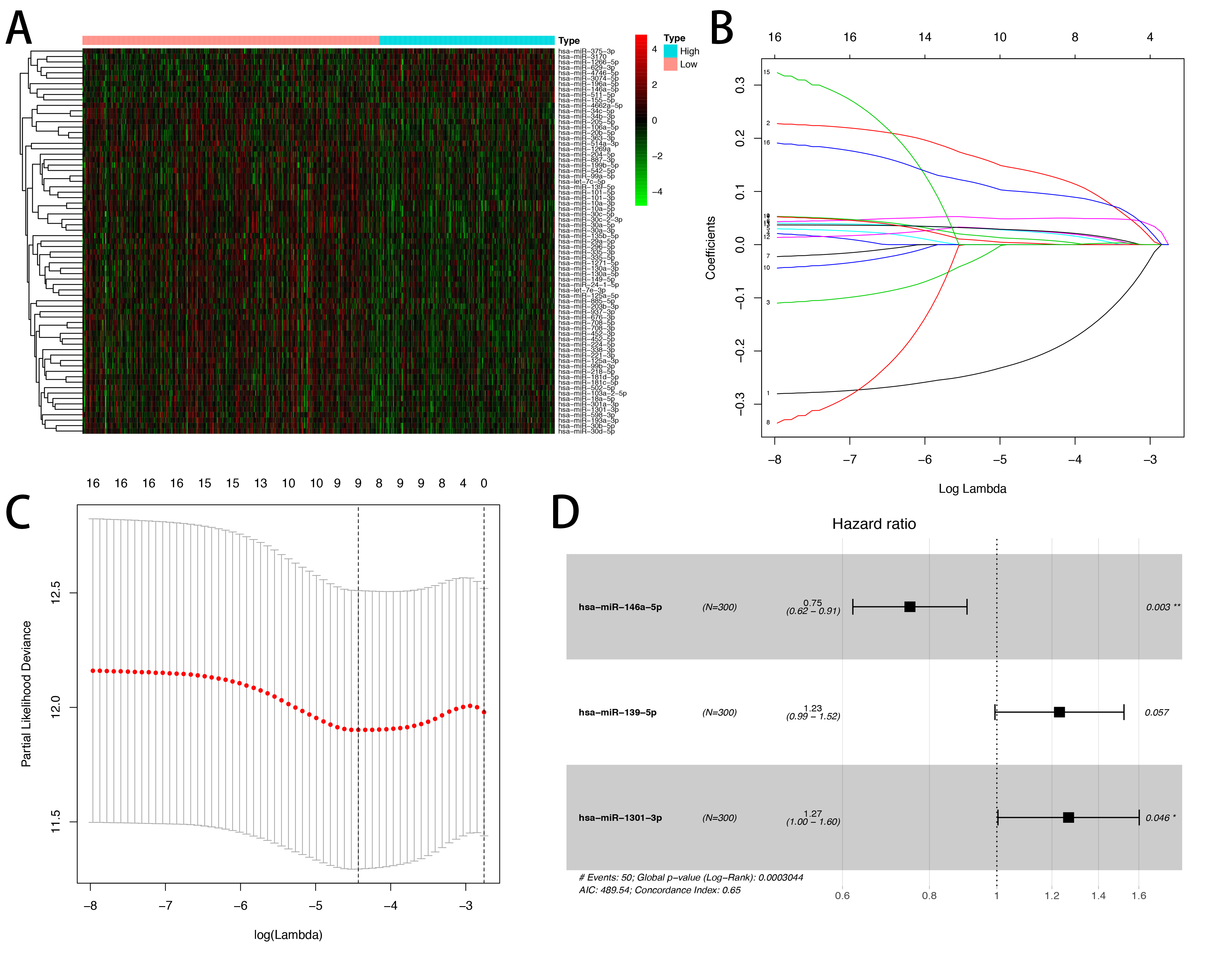

Supplement: Supplemental Material [file KBIE_A_1947940_SM6951.zip › supplementary/FIG S4.jpg]
